# Upregulation of the lncRNA Meg3 induces autophagy to inhibit tumorigenesis and progression of epithelial ovarian carcinoma by regulating activity of ATG3

**DOI:** 10.18632/oncotarget.15955

**Published:** 2017-03-07

**Authors:** Yin-ling Xiu, Kai-xuan Sun, Xi Chen, Shuo Chen, Yang Zhao, Qing-guo Guo, Zhi-Hong Zong

**Affiliations:** ^1^ Department of Biochemistry and Molecular Biology, College of Basic Medicine, China Medical University, Shenyang 110122, China; ^2^ Department of Gynecology, The First Affiliated Hospital of China Medical University, Shenyang 110001, China

**Keywords:** lncRNA, Meg3, autophagy, epithelial ovarian carcinoma, tumorigenesis and progression

## Abstract

Maternally expressed gene 3 (Meg3), a long non-coding RNA, has been reported to be associated with the pathogenesis of multiple malignancies. However, little is known regarding the role of Meg3 in epithelial ovarian cancer (EOC). In this study, we found that the expression of Meg3 was lower in epithelial ovarian carcinoma, and has potential to be considered as a biomarker for ovarian cancer. After transfecting the ovarian cancer cell lines OVCAR3 and A2780 with Meg3, phenotypic changes and autophagy-related molecules were examined. Upregulation of Meg3 inhibited cell proliferation, plate colony formation, induced cell cycle arrest in G2 phases, and promoted apoptosis. Observation of autophagosomes was performed by transmission electron microscopy. The expression levels of LC3-II, ATG3, LAMP1 were elevated, while SQSTM1/p62 expression declined. Upregulated expression of Meg3 also suppressed tumorigenesis *in vivo* in a xenograft mouse model through upregulating ATG3 expression. RIP (ribonucleoprotein immunoprecipitation) and RNA pull-down assays showed that Meg3 was co-immunoprecipitated with ATG3. In addition, Meg3 protected ATG3 mRNA from degradation following treatment with actinomycin D. Overall, our results suggest that the lncRNA Meg3 acts as a tumor suppressor in EOC by regulating ATG3 activity and inducing autophagy.

## INTRODUCTION

Epithelial ovarian carcinoma (EOC) remains one of the most common gynecologic malignancies, with 21,290 new cases diagnosed and 14,000 deaths reported in the United States in 2015 [1]. EOC is often diagnosed at an advanced stage with widely disseminated intra-peritoneal metastasis, and the 5-year survival rate is often below 30% [2]. It is, therefore, essential to identify early stage biomarkers and establish the molecular mechanisms of tumorigenesis and progression.

Long non-coding RNAs (lncRNAs) are a family of non-protein-coding RNAs greater than 200 nucleotides in length [3]. LncRNAs play regulatory and structural roles in allelic expression, and many other physiological and pathological processes [4]. They have been reported to act as tumor suppressors, oncogenes, or both, in various human cancers [5]. One such lncRNA gene is maternally expressed 3 (Meg3), located on chromosome 14q32 [6]. It is an imprinted gene that has been predicted to act as a tumor suppressor involved in the pathogenesis and progression of cancers [7]. A decrease in Meg3 expression has been reported in various human tumors [8–12]. To date, the role of Meg3 in tumorigenesis and the development of EOC and the mechanisms through which it acts remain unknown.

## RESULTS

### Expression of Meg3 is associated with tumorigenesis and progression of EOC

**Figure 1:**
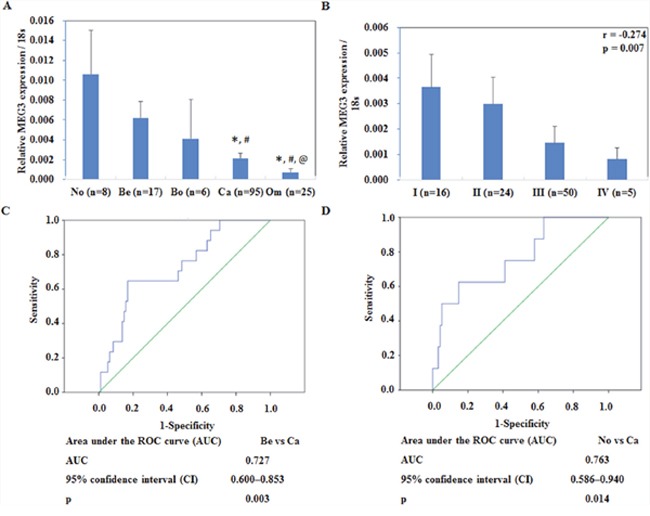
Correlation of lncRNA meg3 expression with pathogenesis and aggressiveness of endometrial carcinoma Meg3 was downregulated in primary ovarian cancer tissues than normal ovarian tissues and benign ovarian tumors **(A)**. Meg3 expression in metastatic omentum was lower than normal ovarian tissues, benign ovarian tumors, and borderline tumor. Expression of Meg3 had a negative correlation with FIGO stages **(B)**. ROC plots for MEG3 for **(C)** benign tumor vs ovarian cancer, **(D)** normal vs ovarian cancer. Abbreviations: No = normal ovarian tissues; Be = benign ovarian tumors; Bo = borderline ovarian tumors; Ca = ovarian cancer; Om = metastatic omentum. * vs. normal ovarian tissues; ^#^ vs. benign ovarian tumors; ^@^ vs. borderline tumor.

Meg3 mRNA expression was lower in EOC tissue as compared to normal ovarian tissues and benign ovarian carcinomas. In metastatic omentum tumors, expression was found to be significantly lower as compared to all above (Figure 1A, P < 0.05), details could be found in Supplementary Table 1. Besides, Meg3 expression has a negative correlation with FIGO stages (Figure 1B, r=-0.274, P < 0.05), details could be found in Supplementary Table 2. ROC analysis results showed that ROC plots for MEG3 for benign tumor vs ovarian cancer, normal vs ovarian cancer AUCs of 0.727, and 0.763, respectively (Figure 1C, 1D, P < 0.05).

### Upregulation of Meg3 expression significantly suppresses cell proliferation

**Figure 2:**
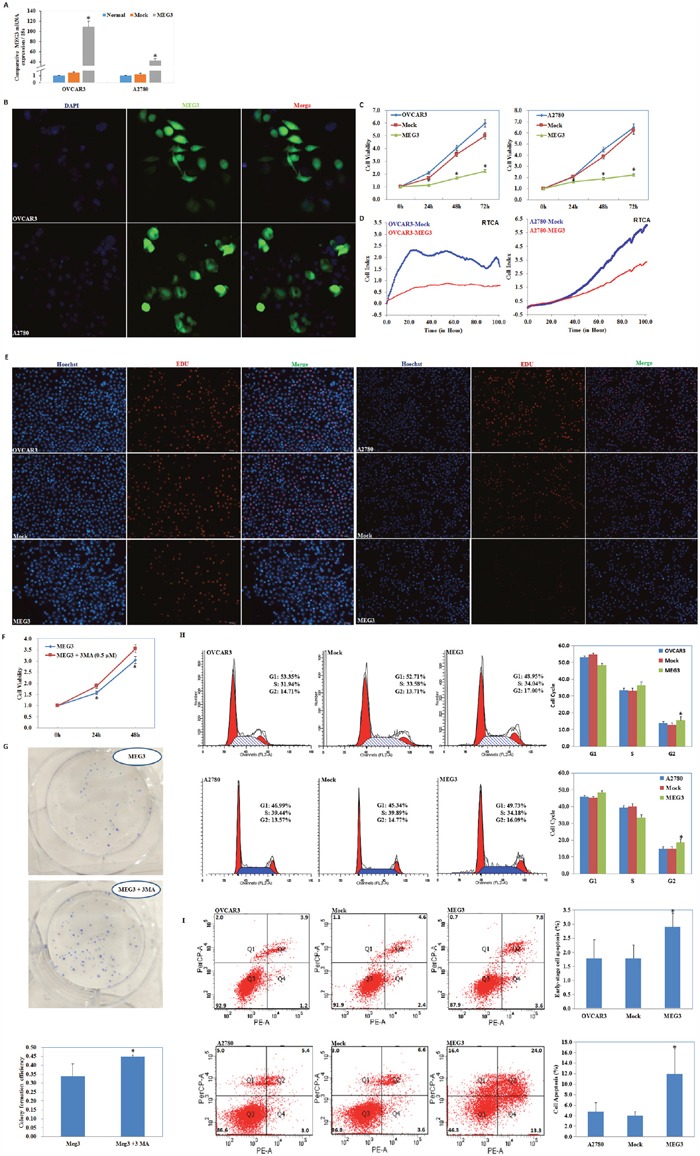
meg3 overexpression inhibited ovarian carcinoma cell proliferation QRT-PCR and Immunofluorescence assay was used to detect meg3 plasmid transfection efficiency **(A, B)**. Meg3 overexpression showed decline of cell viability in MTT **(C)**, RTCA **(D)** and EDU **(E)**. MTT and plate colony formation assay revealed that when given autophagy inhibitor 3MA, Meg3-transfected cells showed improved cell viability **(F)** and colony formation rate **(G)**. Meg3 overexpression showed induction of G2 phase arrest **(H)** and early-stage apoptosis **(I)** compared with control and mock-transfected cells. Results are representative of three separate experiments; data are expressed as the mean ± standard deviation, *P < 0.05.

As determined by qRT-PCR and fluorescence microscopy, Meg3 expression increased after plasmid transfection (Figure 2A, 2B). MTT and RTCA assays were used to determine cell viability. We observed a significant decrease in cell proliferation after upregulation of Meg3 expression (P < 0.05; Figure 2C, 2D). EdU staining demonstrated lower rates of proliferation in the Meg3 overexpression group (Figure 2E). MTT and plate colony formation assay revealed that when given Meg3-transfected cells with autophagy inhibitor 3MA, the cell viability and colony formation rate was improved as compared with Meg3-transfected cells (P < 0.05; Figure 2F, 2G).

### Meg3 overexpression results in G2 phase arrest and an increase in early-stage cell apoptosis of ovarian cancer cells

Analysis by flow cytometry demonstrated that upregulation of Meg3 increased the percentage of cells in the G2 phase compared with control and mock-transfected cells (P < 0.05; Figure 2H). Upregulation of Meg3 clearly induced higher levels of apoptosis 48 h after transfection of Meg3 (Figure 2I, P < 0.05).

### Meg3 overexpression induces autophagy in EOC cells

**Figure 3:**
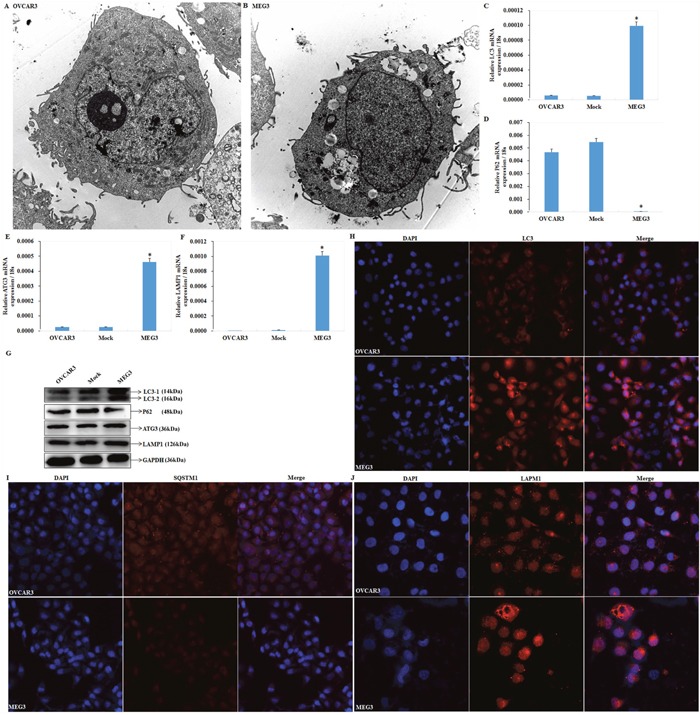
meg3 overexpression regulates ATG3, LC3, SQSTM1, LAMP1 mRNA or protein expression Meg3 overexpression induced autophagosomes formation compared with normal ovarian cancer cell **(A, B)** under transmission electron microscope, induced LC3 **(C)**, ATG3 **(E)**, and LAMP1 expression **(F)**, while declined SQSTM1 expression **(D)** by qRT-PCR and western blotting **(G)**, the accumulation of fluorescent LC3 **(H)** and LAMP1 **(J)** puncta and decline of SQSTM1/P62 **(I)** compared with the negative control. *, P < 0.05.

We observed autophagosome formation in cells transfected with Meg3 by transmission electron microscope (Figure 3A, 3B). An increase in LC3 (Figure 3C), ATG3 (Figure 3E), LAMP1 (Figure 3F) and degraded SQSTM1/p62 (Figure 3D) was demonstrated by qRT-PCR and Western blotting (Figure 3G) after upregulating Meg3. Accumulation of LC3 (Figure 3H), and LAMP1 (Figure 3J) fluorescent puncta was observed following Meg3 transfection in EOC cell. Furthermore, a decrease in SQSTM1 fluorescent puncta was also observed in Meg3 transfection. (Figure 3I).

### Upregulated Meg3 expression suppresses tumorigenesis of epithelial ovarian carcinoma *in vivo*

**Figure 4:**
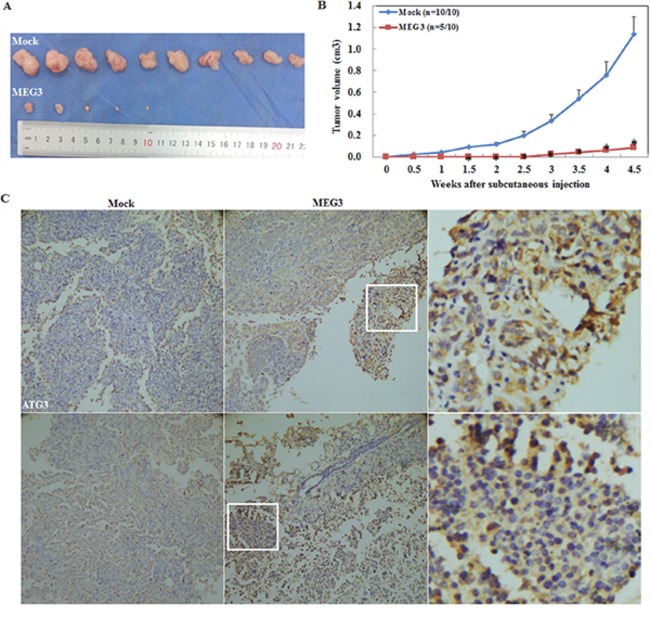
Meg3 overexpression reduced the tumourigenicity of ovarian carcinoma cells *in vivo* Meg3 transfection reduced tumourigenicity after inoculation compared with the control group **(A)** and showed a smaller tumour volume **(B)**. Meg3 overexpression induced ATG3 and LC3 expression compared with the control group **(C)**.

A nude mouse xenograft assay was performed and the results demonstrated that injection of mice with Meg3-transfected cells resulted in tumors with smaller volumes as compared to the control group, as observed within a similar time frame. (P < 0.05; Figure 4A, 4B). IHC analysis showed a significantly higher ATG3 and LC3 expression in the Meg3 overexpression group compared with the control group (Figure 4C).

### Meg3 is co-immunoprecipitated with ATG3

**Figure 5:**
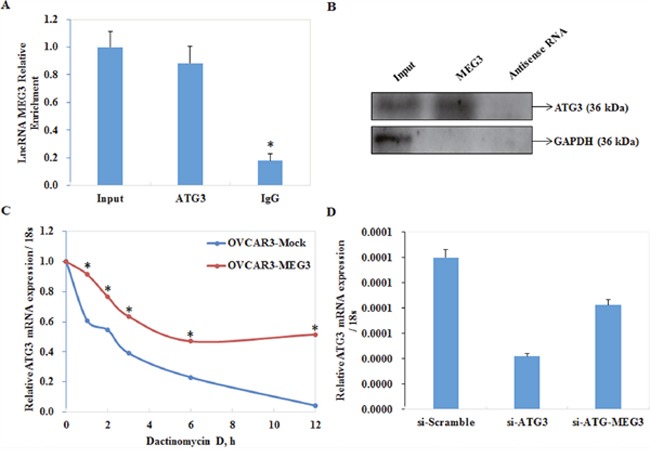
Meg3 was co-immunoprecipitated with ATG3 and protected ATG3 from decay Ribonucleoprotein-immunoprecipitation (RIPs) assay demonstrated an enrichment of lncRNA meg3 **(A)**.*, P < 0.05. RNA pull-down assay showed that ATG3 interacting with biotin-labeled MEG3 was higher than that with antisense of MEG3 group **(B)**. mRNA of ATG3 was relatively resistant to the inhibitor treatment compared with the control group **(C)**. There is no significant difference between si-ATG3 and co-infection of si-ATG3 and Meg3 **(D)**.

Ribonucleoprotein-immunoprecipitation (RIP) assays were performed to confirm the functional interaction between MEG3 and autophagic protein ATG3. RNA from RIP assays with an antibody against ATG3 was used for qPCR analysis, which demonstrated an enrichment of the lncRNA Meg3 (Figure 5A). Next, we examined whether MEG3 interacted with ATG3 protein by RNA pull down, we found that the expression level ATG3 interacting with biotin-labeled MEG3 was higher than that with antisense of MEG3 group (Figure 5B). These data suggested the interaction between MEG3 and ATG3 protein.

### Meg3 prevents decay of ATG3 mRNA

Cells transfected with Meg3 were treated with actinomycin D to study the stability of ATG3 mRNA. Our results showed that ATG3 mRNA in cells transfected with Meg3 showed a slower rate of decay compared with control cells (Figure 5C).

### Downregulation of ATG3 inhibits autophagy in EOC cells

**Figure 6:**
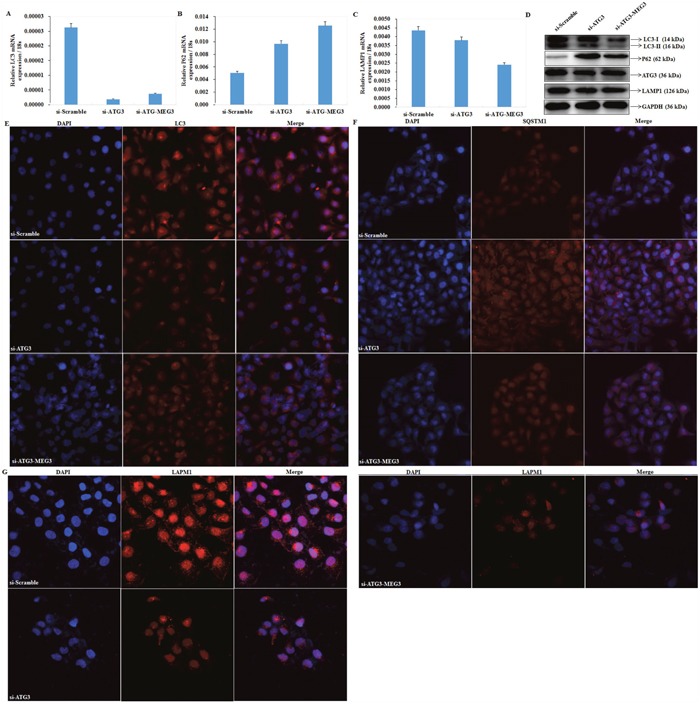
Downregulation of ATG3 regulates LC3, SQSTM1 /P62, and LAMP1 The mRNA expression of LC3 **(A)** and LAMP1 **(C)** were significantly lower in transfection of si-ATG3 than in the control, whereas expression of SQSTM1/P62 **(B)** was induceded. Si-ATG3 and Meg3 co-transfection has no significant difference with si-ATG3 transfection alone. The same results were demonstrated by and western blot **(D)** and Immunofluorescence **(E, F, G)**.

qRT-PCR and Western blot analysis were used to measure LC3, SQSTM1/P62, LAMP1 mRNA and protein expression after transfecting si-RNA to silence expression of ATG3 in OVCAR3 cells. Both mRNA and protein expression of LC3 (Figure 6A) and LAMP1 (Figure 6C) were significantly lower, and expression of SQSTM1/P62 (Figure 6B) was significantly increased compared with the negative control (P < 0.05; Figure 6D). Results of immunofluorescence assays demonstrated that si-ATG3 transfection inhibited LC3 (Figure 6E) and LAMP1 (Figure 6G) accumulation while induced expression of SQSTM1 (P62) (Figure 6F). Besides, increased expression of Meg3 could not reverse effects of si-ATG3 (Figure 5D, Figure 6E, 6F, 6G).

## DISCUSSION

Recently, a series of studies have focused on the molecular mechanism of pathogenesis and progression of tumors, and the action of lncRNAs has attracted intense research interest. Studies have revealed that lncRNAs are involved in various processes including development, cell proliferation, metastasis, fate decision, invasion and migration [13–15], which makes lncRNAs potential diagnostic and prognostic biomarkers, as well as targets for cancer treatment. Meg3, a member of the lncRNA family, plays an important role in multiple tumors. Meg3 is frequently silenced in urothelial cancer tissues and cell lines [16]. Meg3 inhibits the development and progression of colorectal cancer by regulating cell proliferation [17]. In prostate cancer cells, Meg3 promotes apoptosis and acts as a tumor suppressor [18]. Furthermore, Meg3 is downregulated in nonfunctional pituitary adenoma, and the decreased levels are associated with increased tumor invasive abilities [19]. Peng and colleagues demonstrated that the lncRNA Meg3 could inhibit gastric carcinogenesis and serve as a potential target for antineoplastic therapies [20]. In addition, Meg3 was identified as a suppressive gene in lung cancer, cervical cancer and hepatocellular carcinoma [21–23].

To investigate the action of Meg3 in EOC, we analyzed the mRNA expression of Meg3 in normal epithelial ovarian tissues, benign tumor tissues, primary ovarian carcinomas, and metastatic omentum tumors. We found that the expression of Meg3 was reduced significantly in primary tumors as compared to normal ovarian tissue and benign tumors. Furthermore, the expression of Meg3 in metastatic omentum tumors was even lower than in primary ovarian carcinoma. These results demonstrated that there is a close relationship between Meg3 and tumorigenesis, as well as the development of ovarian cancer.

Transfection of ovarian cancer cell lines with Meg3 resulted in inhibition of cell proliferation, colony formation, migration and metastasis, and promotion of G2 phase arrest, as well as cell apoptosis, while after given Meg3-transfected cells with autophagy inhibitor 3MA, the cell viability and colony formation rate was improved as compared with Meg3-transfected cells. Nude mice xenograft assays showed that overexpression of Meg3 could reduce tumorigenesis of ovarian cancer cells *in vivo*. In conclusion, Meg3 plays a suppressive role in EOC, which is consistent with the role of Meg3 in gastric cancer, cervical cancer, lung cancer and hepatocellular carcinoma.

Furthermore, autophagosomes observed under transmission electron microscopy indicated that Meg3 induced autophagy in EOC. Autophagy, in general, is a survival process. Autophagic (type II) cell death usually represents a failed attempt to overcome lethal stress, and disruption of this process promotes rather than inhibits cell death in many cases [24, 25]. Several mechanisms have been proposed to explain the tumor suppressive function of autophagy. [26] It is a multiple-stage process regulated by a number of autophagy-related proteins (Atgs). The microtubule associated protein 1 light chain 3(LC3-I) is conjugated to phosphatidylethanolamine (PE) to form LC3-II, and elevated expression of LC3-II can be used as a biomarker for autophagy. Four residues of ATG3 whose side chains make contacts with ATG12 are important for LC3 lipidation [27]. Upon maturation, LC3-II binds with the LC3-interacting motif of p62, a selective autophagy adaptor for degradation of ubiquitinated substrates to form autophagosomes [28]. The autophagosomes fuse with lysosomes to form autolysosomes, which can be identified by the lysosomal-associated membrane protein 1 (LAMP1), a lysosomal surface marker. During the process of autophagic flux, autophagosomes and their contents, including p62, are digested. If expression of p62 does not decrease, this can be an indication that the progression of autophagy might be stalled [29]. Our qRT-PCR and Western blot results showed an elevation of LC3 and LAMP1, demonstrating that upregulated Meg3 induced autophagy in EOC. Reduced expression of p62 revealed that autophagy was in fact occurring. The Atg-related protein ATG3 was upregulated after transfection with Meg3. Changes in the accumulation of fluorescent puncta also supported these results. In addition, overexpression of Meg3 reduced tumorigenesis and progression of EOC *in vivo* and *in vitro*. Therefore, we suggest that Meg3 acts as a tumor suppressor in EOC, which may be achieved by initiating autophagy and causing type II cell death.

Recent studies have provided a significant perspective on the important role of lncRNAs in regulation of gene expression. Reports have revealed that lncRNAs may act as either scaffolds that contain distinct protein-interacting domains to bring specific protein into proximity of each other, and form unique functional complexes, or as guides to recruit proteins [30–32]. Upregulated expression of Meg3 resulted in elevated expression of LC3, LAMP1, and ATG3, as well as a decrease in p62. Our RIP and RNA pull-down assays revealed direct cross-talk between Meg3 and ATG3. In addition, increased expression of Meg3 was also found to stabilize and inhibit decay of ATG3 mRNA following actD treatment. Transfection of Meg3 after silencing expression of ATG3 with si-RNA, however, did not lead to a significant accumulation of LC3. Therefore, we suggest that the lncRNA Meg3 may interact with ATG3 to form a complex and promote its expression, and autophagy induced by Meg3 is dependent on the presence of ATG3.

In conclusion, the present study has shown for the first time that upregulated expression of Meg3 triggers autophagic flux through an interaction with ATG3 to suppress tumorigenesis and progression of EOC. Meg3 may serve as a potential biomarker of EOC. Our findings may provide a novel insight into the early diagnosis and treatment of ovarian cancer. And further research should delineate the role and regulation of the crosstalk between Meg3 and autophagy in pathogenesis and progression of EOC.

## MATERIALS AND METHODS

### EOC specimens

EOC tissues (Ca), metastatic omentum tissues (Om), borderline tumor (Bo) tissues, benign tumor tissues (Be) and normal ovarian specimens (No) were obtained from patients who underwent surgical resection at the Department of Gynecology, the First Affiliated Hospital of China Medical University (Shenyang, Liaoning, China) among 2005.01-2015.12. Tumor specimens were reviewed by microscopy by two independent pathologists and staged according to the FIGO staging system. Samples were frozen in liquid nitrogen immediately and stored at − 80°C until use. No patients underwent chemotherapy or radiotherapy treatment prior to surgery. Informed consent was obtained from all subjects. Ethics board approval was obtained from the China Medical University Ethics Committee, and all specimens were handled and anonymized in an ethical and legal manner.

### Cell culture and transfection

The human ovarian carcinoma cell lines OVCAR3 and A2780 were obtained from the Tumor Cell Bank of the Chinese Academy of Medical Science (Peking, China). OVCAR3 cells were cultured in RPMI 1640 (HyClone, Logan, UT, USA) and A2780 cells were cultured in Dulbecco's Modified Eagle's Medium (DMEM; HyClone, Logan, UT, USA) supplemented with 10% fetal bovine serum (FBS), and penicillin/streptomycin (100 U/mL). The cells were cultured in an incubator at 37°C and an atmosphere of 5% CO_2_. The medium was changed every one to two days based on the culture state. MEG3-specific pcDNA overexpression vector (pcDNA-MEG3), ATG3-specific siRNA (si-ATG3) and corresponding control including empty pcDNA, and si-scramble were completed by Shanghai Genechem Co., Ltd., China. These recombinants were transfected into OVCAR3 or A2780 cells using Lipofectamine 2000 reagent (Invitrogen, USA) according to the manufacturer's instructions. The sequence of Meg3 plasmid is CTCGAGCTAGCCCCTAGCGCAGACGGCGGAGAGCAGAGAGGGAGCGCGCCTTGGCTCGCTGGCCTTGGCGGCGGCTCCTCAGGAGAGCTGGGGCGCCCACGAGAGGATCCCTCACCCGGGTCTCTCCTCAGGGATGACATCATCCGTCCACCTCCTTGTCTTCAAGGACCACCTCCTCTCCATGCTGAGCTGCTGCCAAGGGGCCTGCTGCCCATCTACACCTCACGAGGGCACTAGGAGCACGGTTTCCTGGATCCCACCAACATACAAAGCAGCCACTCACTGACCCCCAGGACCAGGATGGCAAAGGATGAAGAGGACCGGAACTGACCAGCCAGCTGTCCCTCTTACCTAAAGACTTAAACCAATGCCCTAGTGAGGGGGCATTGGGCATTAAGCCCTGACCTTTGCTATGCTCATACTTTGACTCTATGAGTACTTTCCTATAAGTCTTTGCTTGTGTTCACCTGCTAGCAAACTGGAGTGTTTCCCTCCCCAAGGGGGTGTCAGTCTTTGTCGACTGACTCTGTCATCACCCTTATGATGTCCTGAATGGAAGGATCCCTTTGGGAAATTCTCAGGAGGGGGACCTGGGCCAAGGGCTTGGCCAGCATCCTGCTGGCAACTCCAAGGCCCTGGGTGGGCTTCTGGAATGAGCATGCTACTGAATCACCAAAGGCACGCCCGACCTCTCTGAAGATCTTCCTATCCTTTTCTGGGGGAATGGGGTCGATGAGAGCAACCTCCTAGGGTTGTTGTGAGAATTAAATGAGATAAAAGAGGCCTCAGGCAGGATCTGGCATAGAGGAGGTGATCAGCAAATGTTTGTTGAAAAGGTTTGACAGGTCAGTCCCTTCCCACCCCTCTTGCTTGTCTTACTTGTCTTATTTATTCTCCAACAGCACTCCAGGCAGCCCTTGTCCACGGGCTCTCCTTGCATCAGCCAAGCTTCTTGAAAGGCCTGTCTACACTTGCTGTCTTCCTTCCTCACCTCCAATTTCCTCTTCAACCCACTGCTTCCTGACTCGCTCTACTCCGTGGAAGCACGCTCACAAAGGCACGTGGGCCGTGGCCCGGCTGGGTCGGCTGAAGAACTGCGGATGGAAGCTGCGGAAGAGGCCCTGATGGGGCCCACCATCCCGGACCCAAGTCTTCTTCCTGGCGGGCCTCTCGTCTCCTTCCTGGTTTGGGCGGAAGCCATCACCTGGATGCCTACGTGGGAAGGGACCTCGAATGTGGGACCCCAGCCCCTCTCCAGCTCGAAATCCCTCCACAGCCACGGGGACACCCTGCACCTATTCCCACGGGACAGGCTGGACCCAGAGACTCTGGACCCGGGGCCTCCCCTTGAGTAGAGACCCGCCCTCTGACTGATGGACGCCGCTGACCTGGGGTCAGACCCGTGGGCTGGACCCCTGCCCACCCCGCAGGAACCCTGAGGCCTAGGGGAGCTGTTGAGCCTTCAGTGTCTGCATGTGGGAAGTGGGCTCCTTCACCTACCTCACAGGGCTGTTGTGAGGGGCGCTGTGATGCGGTTCCAAAGCACAGGGCTTGGCGCACCCCACTGTGCTCTCAATAAATGTGTTTCCTGTCTTAACAAAAAGGATCC. The sequence of the siRNA targeting ATG3 was as follows Sense: CAUUGAGACUGUUGCAGAAdtdt; Anti-sense:UUCUGCAACAGUCUCAAUGdtdt.

### MTT proliferation assay

Three thousand cells were seeded into each well of 96-well plate in 100 μL medium. At the time points of 0 h, 24 h, 48 h and 72 h, 20 μL of 5 mg/mL MTT reagent was added and plates incubated for 4 h. The medium was then removed and replaced with 150 μL DMSO. Absorbance at 490 nm was measured using a microplate spectrophotometer (Bio-Tek Instruments, Winooski, VT).

### Real-time cell analyzer (RTCA) assay

For real-time cell proliferation assays, 50 μL medium was added to each well of a 96-well E-Plate for background measurement. Subsequently, 5 × 10^3^ cells in 100 μL medium were seeded into each well of the E-Plate. Following incubation at room temperature for 30 min, the E-Plates containing cells were placed on the RTCA SP/MP station positioned in a cell culture incubator. The CI values were measured automatically every 15 minutes to form a continuous proliferation curve.

### Cell cycle analysis

After washing with PBS, cells were treated with trypsin to be collected and washed, then fixed with 70% ice-cold ethanol at -20°C for at least 12 h. Cells were washed and re-suspended by centrifugation then stained with PI following the manufacturer's protocol (BD Biosciences, New Jersey, USA) for cell cycle analysis by flow cytometry.

### Plate clone formation assay

Cells were trypsinized, counted, suspended and seeded into six-well plates at a density of 500 cells per well, in regular culture medium, then cultured at 5% CO2 and 37°C for 2 weeks until visible clones appeared. The medium was discarded and the cells were carefully washed twice with PBS. After being fixed with methanol for 15 min, the cells were stained with Giemsa's solution for 15 min before washing with tap water and air-drying. The clone formation rate was calculated with the formula: Plate clone formation efficiency = (number of clones/number of cells inoculated) × 100%. All the experiments were repeated 3 times and the average values were reported.

### Apoptosis assays

Cells were collected and centrifuged for 5 min and washed twice with cold PBS. Cells were then resuspended in 100 μL 1×buffer with 5 μL 7AAD and 5 μL PE-labeled annexin V (KeyGen) per sample in the dark. An additional 400 μL of 1× buffer was added and the rate of apoptosis for each samples determined by flow cytometry within 1 h.

### RT-PCR

TRIzol (Takara, Shiga, Japan) was used to extract the total RNA from EOC cell lines and tissues. Isolated RNA was transcribed into cDNA using an avian myeloblastosis virus reverse transcriptase and random primers (Takara, Shiga, Japan). Amplification of the cDNA was performed by real-time quantitative PCR using the SYBR Premix Ex Taq™ II kit (Takara, Shiga, Japan). The expression level of each target gene was normalised to 18s mRNA. The data analysis was calculated according to the sample threshold cycle (Ct) value from three independent experiments, primers for RT-PCR could found in Supplementary Table 3.

### Western blotting

The complete proteome from ovarian cancer cells was extracted in RIPA buffer, preventing protease-mediated degradation of samples. Protein concentration was determined for each sample, and 40 μg of the denatured proteome was resolved by 15% SDS-polyacrylamide gel electrophoesis and then electro-transferred to Hybond membranes (Amersham, Munich, Germany). After blocking the membranes with 5% fat-free milk at room temperature for 2 h, membranes were incubated with the primary antibodies primary antibodies targeting ATG3, LAMP1 (1:1000, Proteintech, Proteintech Group, USA), LC3 (1:2000, MBL, Medical & Biological Laboratories Co., LTD, USA), and SQSTM1 (1:1000, Bioss, Peiking, China) at 4°C overnight. Membranes were washed three times with TBST then secondary antibodies (anti-rabbit), at dilutions of 1:5000 were added. Following incubation for 2 h at room temperature, the protein bands were visualized by enhanced chemiluminescence (ECL) following the manufacturer's instruction (Santa Cruz Biotechnology, Santa Cruz, CA, USA). GAPDH (Proteintech, Proteintech Group, USA) served as the loading control.

### RNA pull-down

The interaction between LncRNA MEG3 and ATG3 protein was examined using Pierce Magnetic RNA-Protein Pull-Down Kit (Thermo fisher) according to the manufacturer's protocols. Biotin-labeled MEG3 or antisense RNA was co-incubated with protein extract of OVCAR3 cells and magnetic bead. The generated bead-RNA-Protein compound was collected by low-speed centrifugation. After washed with Handee spin columns, bead compound was boiled in SDS buffer, and the retrieved protein was detected by western blot with expressional level of GAPDH as control.

### RNA Immunoprecipitation (RIP) assay

The Magna RIP RNA-Binding Protein Immunoprecipitation Kit (Millipore, Bedford, MA, USA) was used for RIP assays following the manufacturer's protocol. Briefly, OVCAR3 cells were collected and lysed using RIP lysis buffer. Whole-cell extracts were incubated with RIP buffer containing magnetic beads conjugated to human anti-ATG3 antibody or the control IgG. Proteinase K was added to the samples to digest the protein, and the immunoprecipitated RNA isolated. Purified RNA was used for qRT-PCR analysis. Expressional level of GAPDH was as control.

### Actinomycin D (actD) assay

Control and transfected cells were treated with actD (dissolved in 100% ethanol) at a final concentration of 1-5 μM. The actD was added to cells 0 h, 1 h, 2 h, 4 h, 6 h or 12 h prior to RNA extraction with TRIzol reagent. Subsequently, qRT-PCR was used to analyze changes in RNA levels.

### Xenografts assays

All animal studies were approved by the China Medical University Animal Care and Use Committee, and all experimentation on animals was performed in agreement with the National Institutes of Health Guide for the Care and Use of Laboratory Animals. Four-week-old female BALB/c nude mice with weights of approximately 20 g were obtained from Vital River Laboratories (VRL; Beijing, China) and housed in a pathogen–free and temperature-controlled environment. Two hundred microliters of PBS containing approximately 1 × 10^7^ (A2780, transfected with mutated or wildtype Meg3) were injected into the right flanks of the mice. The tumour volume was directly measured following inoculation and weight calculated using the formula: (length × width^2^)/2.

### Immunohistochemistry (IHC)

Paraffin-embedded tissue sections were deparaffinized in xylene and rehydrated in a graded series of ethanol solutions and then incubated for 20 min in 3% H_2_O_2_ to endogenous peroxidase activity. Next, the sections were heated in target retrieval solution (Dako) for 15 min in a microwave oven (Oriental Rotor) to retrieve the antigen. Non-specific binding was blocked by incubation with 10% goat serum for 2 h at room temperature. Slides were then incubated overnight at 4°C with anti-ATG3 primary antibody. An appropriate secondary antibody was added and incubated for 20 min at 37°C, and binding was visualized with 3,39-diaminobenzidine tetrahydrochloride (DAB). After each treatment, slides were washed three times with TBST for 5 min. Three independent observers (CS, YZ and ZZH) randomly selected and counted 100 cells from five representative fields from each section. Any discrepancies were checked by three observers until a consensus was reached. Positive expression was graded as follows: 0 = negative; 1 = 1%-50%; 2 = 51%-74%; 3 ≥ 75%. The staining intensity was graded as follows: 1 = weak; 2 = intermediate; 3 = strong. The two grades were multiplied to obtain a final score: - = 0; + = 1-2; ++ = 3-4; +++ = 6-9).

### Immunofluorescence assay (IFA)

Transfected or control OVCAR3 cell slides were fixed with 4% formaldehyde, blocked using 5% skimmed milk in 0.01 M PBS, pH 7.2 (PBS-M). Primary antibodies targeting LC3, SQSTM1 (P62) or ATG3 (Sigma-Aldrich, St Louis, MO, USA), were diluted 1:500 in PBS and added to the slides. After incubation overnight at 4°C, slides were washed once with 0.2% Tween 20 in PBS and twice with PBS, and then incubated for 30 min at room temperature with anti-rabbit IgG–TRITC (1:100, Santa Cruz Biotechnology). The slides were washed three times with PBS, then fixed with glycerol/PBS (2:1, pH 9.0), and observed and photographed using a laser confocal microscope (Olympus, Tokyo, Japan).

### Statistical analysis

Data were analyzed using SPSS 17.0 software (SPSS Inc., Chicago, IL, USA), and are presented as mean ± SD. Two groups were compared using an unpaired, two-tailed Student's t-test. Spearman rank correlation was used to test the association between Meg3 expression and FIGO stages. The ROC characteristics curve was used to calculate the sensitivity and specificity values of Meg3. and P < 0.05 was considered to be statistically significant.

## SUPPLEMENTARY MATERIALS TABLES


