# Supplementary material for: Upregulation of the lncRNA Meg3 induces autophagy to inhibit tumorigenesis and progression of epithelial ovarian carcinoma by regulating activity of ATG3
Source: Oncotarget. 2017 Mar 7;8(19):31714–25. doi: 10.18632/oncotarget.15955 (PMC5458242; doi:10.18632/oncotarget.15955)
Supplement: Supplementary file 1 [file oncotarget-08-31714-s001.pdf]

Upregulation of the lncRNA Meg3 induces autophagy to inhibit tumorigenesis and progression of epithelial ovarian carcinoma by regulating activity of ATG3

SUPPLEMENTARY TABLES

Supplementary Table 1: LncRNA MEG3 expression in normal ovary and ovarian carcinoma tissues

Groups	N	LncRNA MEG3 expression / 18s	*P* value
Normal Ovary	8	0.0106 ± 0.0124	***4.55E-05***
Benign tumors	17	0.0062 ± 0.0069	***0.002***
Borderline tumors	6	0.0041 ± 0.0096	0.183
Ovarian carcinoma	95	0.0022 ± 0.0048	
Metastatic omentum tissues	25	0.0007 ± 0.0016	0.071

Bold and Italics means P < 0.05.

Supplementary Table 2: Correlation of LncRNA MEG3 expression with different clinicopathological features of ovarian carcinoma

Clinicopathological features	N	LncRNA TDRG1 expression / 18s	*P* value
**The pathology types**			0.100
Serous carcinoma	74	0.0018 ± 0.0046	
The other pathology types	21	0.0034 ± 0.0053	
**Age**			0.288
≤ 53	47	0.0025 ± 0.0053	
> 53	48	0.0019 ± 0.0043	
**FIGO stages**			***0.030***
I-II	40	0.0033 ± 0.0051	
III-IV	55	0.0014 ± 0.0044	
**Pathology classification**			0.331
Well	28	0.0025 ± 0.0045	
Mod + Poor	67	0.0020 ± 0.0049	

Bold and Italics means P < 0.05.

Supplementary Table 3: Primers for RT-PCR

Gene	Primer sequence	Product size (bp)	Extension time (sec)
MEG3	F: 5'- TGCTTCCTGACTCGCTCTA-3'	234	34
	R: 5'- CTTCCATCCGCAGTTCTT-3'		
ATG3	F: 5'-GTCCACCACTGTCCAA-3'	227	34
	R: 5'-GCTTCCGTTATTCCTG-3'		
ATG7	F: 5'- GAACAAGCAGCAAATGA-3'	148	34
	R: 5'- GACAGAGGGCAGGATAG-3'		
LC3	F: 5'- TCGCCGACCGCTGTAA-3'	286	34
	R: 5'- AAGCCGTCCTCGTCTTTCT-3'		
SQSTM1	F: 5'- TGGAGCACGGAGGGAA-3'	304	34
	R: 5'- TCTGGCATCTGTAGGGACTG-3'		
LAMP1	F: 5'- TGACAAGGCTTCTCAACATC-3'	126	34
	R: 5'- CATTCATCCCGAACTGG-3'		
18s	F: 5'- GAAACGGCTACCACATCC-3'	167	34
	R: 5'- ACCAGACTTGCCCTCCA-3'		

AT = annealing temperature.
